# Kinematic Comparison on Lower Limb Kicking Action of Fetuses in Different Gestational Weeks: A Pilot Study

**DOI:** 10.3390/healthcare9081057

**Published:** 2021-08-18

**Authors:** Hairong Chen, Yang Song, Rongrong Xuan, Qiuli Hu, Julien S. Baker, Yaodong Gu

**Affiliations:** 1 Faculty of Sports Science, Ningbo University, Ningbo 315211, China; chenhairong233@163.com (H.C.); huqiuli@nbu.edu.cn (Q.H.); 2 Doctoral School on Safety and Security Sciences, Obuda University, 1034 Budapest, Hungary; 3 Faculty of Engineering, University of Szeged, 6724 Szeged, Hungary; 4 The Affiliated of School of Medicine of Ningbo University, Ningbo 315211, China; 5 Centre for Health and Exercise Science Research, Department of Sport and Physical Education, Hong Kong Baptist University, Hong Kong 999077, China; jsbaker@hkbu.edu.hk

**Keywords:** fetal movement, kicking action, kinematic, simi motion, ultrasound

## Abstract

The fetal movements during different gestational weeks are essential for normal musculoskeletal development. The kinematic characteristics of fetuses with small differences in gestational weeks may be different and important. Ultrasonographic videos of fetal kicking action and plantarflexion action were collected from three healthy pregnant women (24, 27, and 30 gestational weeks) with normal fetal development. The kinematic characteristics, including angular range and angular velocity, were analyzed. These kinematic parameters were measured using simi motion. The final knee angle was found to decrease with progressive gestational weeks. Compared with 24 w, the knee joint angle at 27 w and 30 w was significantly reduced at the end of a kick-type movement (*p* < 0.01). Except for the mean angular velocity of the knee joint, there were no significant differences in the other conditions. The value at 30 w for mean angular velocity was significantly higher than that at 24 w (*p* = 0.02). In the ankle joint, no significant differences were observed between different conditions. Therefore, we can conclude that there was no significant difference in the kinematic characteristics of the ankle joint for small gestational age gaps, but there was a significant difference in the knee joint. As the gestation weeks increase, the range of kicking motion tends to decrease. The reason may be that with the increase of gestational weeks, fetal lower limb musculoskeletal development is gradually enhanced; the slower growth rate indicates that development reaches a peak level in weeks 24 to 30.

## 1. Introduction

Fetal movement during pregnancy is a natural process of fetal growth and development, and fetal movement plays an essential role in fetal musculoskeletal development [1]. Scholars have previously provided research on the movement of the fetus [2,3]. However, few studies have focused on the differences in the kinematic characteristics of fetuses with small intervals between gestational weeks. Studies have found that fetal movement is closely related to the health of the fetus, and a decrease in fetal movement may indicate congenital diseases following birth [4]. Ultrasound is a meaningful direct observation method to observe fetal movement in utero and the gold standard for objective real-time quantification [5].

Previous studies have used a variety of methods to study fetal movement, such as opensim simulation, finite element modeling, finite element methods, and video analysis using autoCAD. Computer simulation combined with finite element is the forefront of research development [6]. The generation of a three-dimensional finite element model of the uterine environment during pregnancy using dynamic MRI of fetal movement and use musculoskeletal modeling technology to estimate fetal joint force has been investigated [7]. Electrical impedance tomography (EIT) based medical imaging using finite element methods (FEM) can also be used to monitor fetal movement [8]. However, these methods are time-consuming and laborious; from modeling to analysis, the process is tedious, and the accuracy of the final model may not be accurate. Kinematic analysis of fetal motion videos by software is a simple and feasible way to understand fetal motion [9].

The German simi motion system is widely used in sports medicine, injury research, rehabilitation, sports movement technology, and biomechanical analysis of gait. Simi motion can achieve 2D and 3D image capture and analysis [10]. It can be used manually or by using automatic mark point recognition and tracking to analyze motion actions. The system uses 25 frames per second for standard recording and can increase to 10,000 frames per second during high-speed recording. Synchronization error is only 1 ms, video playback and motion capture can be performed at any speed, and characteristic gait parameters such as distance, angle, speed, and acceleration can be displayed using graphics [11]. Using simi motion analysis software to analyze movements, which provides accurate and reliable kinematic parameters, data collection is fast and easy to use [12,13]. Previous studies have rarely used the simi motion system to analyze fetal movement in the uterus. This study uses the simi motion system to analyze the ultrasound video of fetal motion, which is a relatively new and novel methodology.

Different factors such as experimental design and data analysis methods for studying fetal movement can easily cause differences in the final features of fetal movement [4]. A more reliable fetal movement trend can be obtained through the descriptive processing and kinematic analysis of fetal movement to evaluate fetal movement ability better and provide baseline data for follow-up research [10]. Previous studies have conducted kinematic analysis on the movement of pregnant women and fetuses in the first, second, and third trimesters of pregnancy. However, the period was too large to describe the differences in the kinematic characteristics of the fetus between the small gestational week gaps. Therefore, it may not be helpful for doctors to judge abnormal movement or diagnosis-related diseases. Without a more precise reference, there is a lack of objective quantification of the characteristics of fetal movement between the small gestational week gaps during specific gestation periods.

Therefore, the purpose of this study is to explore and analyze the kinematic characteristics of normal fetuses with small difference in gestational weeks using simi motion to analyze ultrasound videos.

## 2. Materials and Methods

### 2.1. Participants

This study was approved by the Ethics Committee of Ningbo University. The subjects were recruited in the Affiliated Hospital of Ningbo University, and the recruited pregnant women signed informed consent forms.

Following evaluation by the obstetrician, three pregnant women were selected, and the fetus in the womb was a singleton. Pregnant women were between 27 and 30 years of age and had average amniotic fluid volumes. The specific age and corresponding gestational age were as follows: 24 w: 28 years old, 27 w: 27 years old, and 30 w: 30 years old. The size of fetal growth was consistent with the pregnant woman’s time of pregnancy and corresponded to the biological characteristics of a normal fetus. The selected pregnant women were excluded by medical examination from hypertension, diabetes, preeclampsia, etc. At the same time, the fetus was examined for growth restriction in the uterus and chromosomal abnormalities. Previous studies have shown that fetal leg movements are not related to the sex of the fetus. Therefore, the sex of the fetus was not considered in the selection of subjects [14].The gestational weeks of the three pregnant women were 24 w, 27 w, and 30 w, respectively. Using 2D ultrasound scans, videos of the fetal lower limb extension and plantar flexion were collected.

### 2.2. Ultrasound Scanning Processing

Using 2D ultrasound equipment to scan, the acquired ultrasound video frame rate was 50 frames per second. Pregnant women were asked not to eat within 2 h before the scan, and the ultrasound scan was performed at night [11]. The subjects were required to rest for half an hour before the ultrasound scan and maintained a comfortable lying position during the scan [9]. The scanned fetal motion video was exported in the format of AVI and annotated for researchers to analyze fetal motion.

### 2.3. Kinematics Analysis Processing

The mark points were selected in the simulated movement based on the movements to be analyzed. The hips, knees, and ankles were selected as the marking points for the knee joint stretching exercise. The knee, ankle, and foot were selected as the marking points for the plantar flexion movement to establish the connection. Fetal motion video in AVI format was imported for calibration; the coordinate system was determined. In the first frame of the video, we set up the *x*-axis and *y*-axis to be perpendicular to each other. Then we imported the video again to track. The tracing points and automatic tracking and angle analysis performed by the software was based on this calibration. By denoting the mark points of each frame, the overall trend change of fetal movement was obtained, and then the kinematics data of fetal lower limbs were analyzed and processed by appropriate software.

#### 2.3.1. The Angle of the Knee and Ankle Joints of the Lower Extremities

In each frame of the video, the angle of the knee and ankle joints of the lower limbs of the fetus was recorded by selecting the markers in each frame.

**Figure 1:**
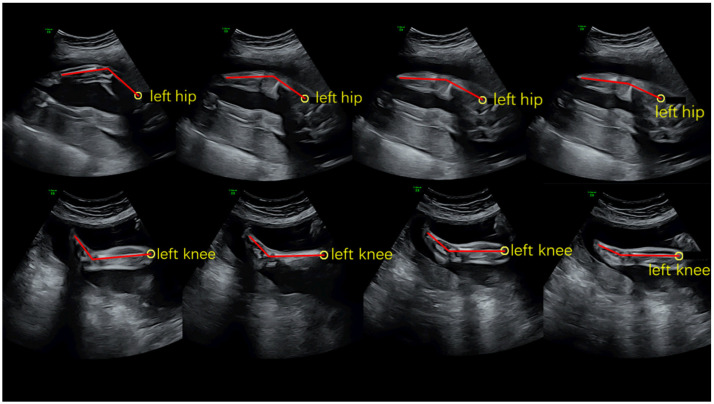
Keyframes during simi motion processing.

Knee extension angle was obtained by measuring the backward angle between thigh segments and the leg segment, and ankle plantarflexion angle was obtained by measuring the anterior angle between leg segments and the foot segment. According to the changes in fetal movements, several keyframes were intercepted. Figure 1 shows the placement of each marker.

#### 2.3.2. The Angle Velocity of the Knee and Ankle Joints of the Lower Extremities

We know the time of each frame and can compute the angle difference by comparing the two frames. We can calculate the corresponding angular velocity of each frame of the knee extension and ankle plantarflexion on the *x*-axis using simi motion.

### 2.4. Statistical Analysis

The data were analyzed using SPSS (Version 25.0, Chicago, IL, USA), and the statistical significance was set to 0.05. The Shapiro–Wilk test was used to verify the normal distribution of variables. One-way ANOVA was performed to determine the kinematic differences between knee extension and ankle plantarflexion of fetuses in different gestational weeks. The analysis included start angle, final angle, start angular velocity, final angular velocity, average angular velocity, and maximum angular velocity.

## 3. Results

### 3.1. Lower Limb Joint Angle Changes

**Figure 2:**
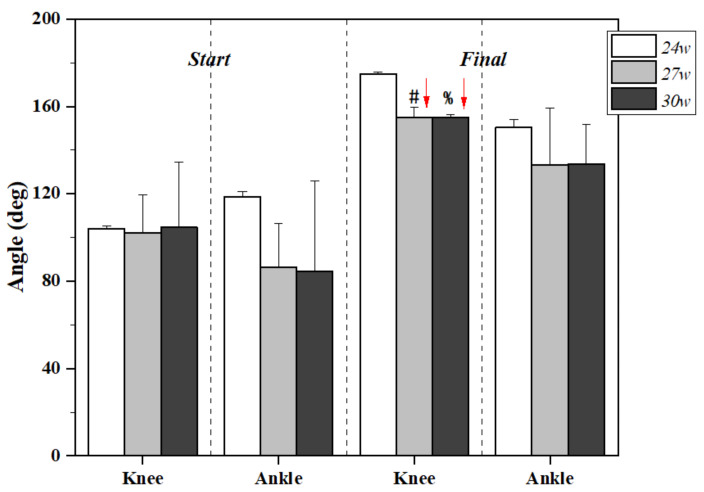
Lower limb joint angle changes during kicking motion at 24, 27, and 30 gestational weeks. Note: # indicates significant differences between 24 w and 27 w; % indicates significant differences between 24 w and 30 w.

The lower limb joint angle changes during kicking motion at 24, 27, and 30 gestational weeks are presented in Figure 2 and Table 1. Although there were no significant differences between gestational weeks in the knee joint during the start position of kicking, the final angle of the knee joint was smaller as the gestational week increased. Both 27 w and 30 w presented significantly smaller knee angles at the end of kicking motion when compared with 24 w (*p* < 0.01). In terms of the ankle joint, however, no significant changes were found among different conditions.

### 3.2. Lower Limb Joint Angular Velocity Changes

**Figure 3:**
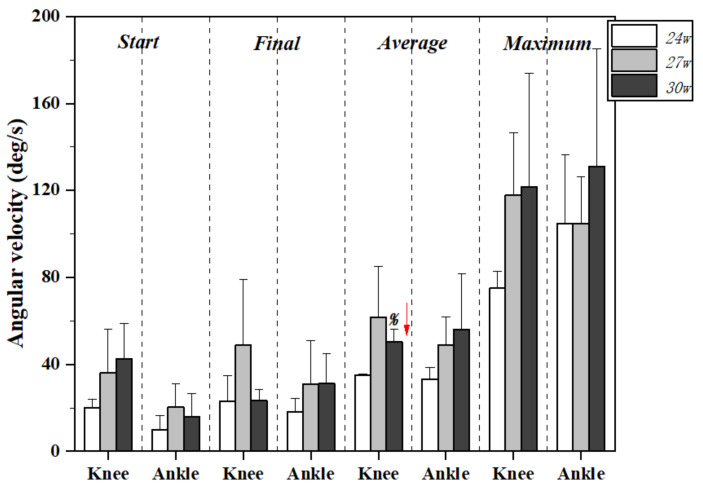
Lower limb joint angular velocity changes during kicking motion at 24, 27, and 30 gestational weeks. Note: % indicates significant differences between 24 w and 30 w.

Figure 3 and Table 2 exhibit the lower limb joint angular velocity changes during kicking motion at 24, 27, and 30 gestational weeks. No significant differences were found among conditions except the average angular velocity for the knee joint, with the value increasing significantly at 30 w compared to 24 w (*p* = 0.02).

## 4. Discussion

There is evidence that there are significant differences in the specific movement patterns of fetuses. In addition, the kinematic parameters of the kick are lacking, thus the aim of this study was to explore and analyze the kinematic characteristics of normal fetuses with small difference in gestational weeks using simi motion to analyze ultrasound videos [15].

By tracking the joint angle of the fetus as it kicks, we noticed no difference in the start angle of the fetus. In addition, there was no difference in the start angle of knee extension and ankle plantarflexion between the three pregnant women. The reason may be that the fetus usually holds the knee and ankle joints at a comfortable angle in the womb [16,17].

The final angle of the knee at 27 weeks and 30 weeks was smaller than that of 24 weeks, and the difference was statistically significant. The knee extension angle of 24 weeks was 174.76 ± 1.04 deg, significantly different from the knee extension angle in 24 weeks and 30 weeks. The angle of fetal knee extension at 27 and 30 weeks decreased to 154.99 ± 4.49 deg and 154.89 ± 1.55 deg, but there was no significant difference between the angle of fetal knee extension at 27 and 30 weeks. In terms of the ankle joint however, no significant changes were found among different conditions. The angle of the ankle joint was deviated enough that, at 24 weeks of the pregnant woman, there was more space for the ankle joint of the fetus in the womb, thus the start angle of the ankle joint was not significantly different in a quiet and relaxed state. At 30 weeks, there was less space available for the ankle joint of the fetus to move. A slight change in the position of the fetus can cause the ankle joint to be restricted, thus there will be a large difference in the ankle joint in a quiet state. The final angle of the ankle was also associated with slight changes in the position of the fetus and the remaining space in the uterus. Thus, we did not obtain a statistical difference in the angle of ankle. In contrast, we found the knee joint final angle to be smaller as the gestational weeks increased.

There was no statistical difference in the angular velocity of knee extension in 24 weeks, 27 weeks, and 30 weeks, except for the mean angular velocity of the knee joint. The mean angular velocity of the knee joint in 24 weeks was 35.04 ± 0.48 deg/s, the mean angular velocity of the knee joint in 27 weeks was 61.49 ± 23.44, and the mean angular velocity of the knee joint in 30 weeks was 50.22 ± 5.83 deg/s. Thus, the difference between the 30 weeks and the 24 weeks is statistically significant, and its value increased significantly at 30 w compared to 24 w (*p* = 0.02).

These results support that the knee extension range of fetal movements decreases in 24 w, 27 w, and 30 w gradually. At the same time, the angular velocity of knee extension increases gradually. However, there is no difference between the starting angle, final angle, starting angular velocity, final angular velocity, average angular velocity, and maximum angular velocity of ankle plantarflexion.

Fetal movement is affected by many factors. It mainly includes two aspects: the change of the fetal physical environment in the womb and the musculoskeletal development of the fetus itself. Both the pregnant women and the fetus were normal and healthy in this research; thus, the main factors affecting fetal movement were the changes in fetal growth, development, and the changes in the fetal physical environment. Changes in the fetal mechanical environment include the amount of amniotic fluid in the uterus, the position and movement of the fetus, and the remaining space in the uterus. Amniotic fluid can make the movement of the fetus unrestricted as it grows. However, as the physical environment changes, the movement of the fetus is restricted. As the fetus grows, the fetus grows more prominent, the amount of amniotic fluid decreases, and the space left in the fetus decreases; thus, the movement of the fetus becomes increasingly restricted [15,18].

The regularity was confirmed by comparing fetal knee extension and ankle plantarflexion at different gestational weeks. The range of the knee joint extension showed a decreasing trend with the increase of gestational weeks. As a result, with the increase of the gestational weeks, the fetal musculoskeletal system, although gradually developed, is limited by reducing the space remaining in the uterus; thus, the range of extension of the knee joint is limited. There was no statistical difference between the final angle of the fetal ankle joint at 24 weeks, 27 weeks, and 30 weeks. The reason may be that the ankle joint occupies less space in the womb. As gestational weeks increase, there is less space in the womb, and the fetus has less room to move; however, there is enough space left for ankle movement.

The angular velocity of the fetal joints can be used to evaluate the impact of cocaine exposure on the fetus [19]. Exposure of pregnant women to drugs before childbirth have specific effects on the fetus [20]. Fetal knee angular velocity can be quantitatively assessed with a high degree of reliability. The experimental results showed that for normal and healthy pregnant women, the mean angular velocity for knee joints between 24 w and 30 w was statistically different. As the gestational weeks increased, the mean angular velocity of the knee joint increased, although there was no significant difference between the start and final kick angular velocity. There was no statistical difference in the fetal ankle angular velocity between different gestational weeks, but the overall trend increased. The mean angular velocity of the knee increased slowly with gestation from 27 to 30 weeks. The increase in angular velocity may be due to musculoskeletal development of the lower extremities becoming stronger as the gestational weeks increase. The growth rate slowed, indicating that development reached a peak level.

This study collected knee extension and ankle plantarflexion movements of pregnant women at 24, 27, and 30 weeks. All previous knee and ankle movement results confirm the environmental and the effects of the biomechanical variables on early motor behavior. Scholars have studied the kinematic characteristics of fetal movement, joint range of motion, and mean velocity during the first, second, and third trimester. There were no statistical differences in range and mean velocity of hip flexion and extension between the three trimesters, but there were statistical differences in these two parameters for knee flexion and extension and ankle plantar flexion and dorsiflexion [4]. This experiment described the kinematic characteristics of the fetus at 24, 27, and 30 weeks. The pregnant women in this experiment had a gestational interval difference of three gestational weeks. Although the overall trend was like previous studies, the differences at small intervals between gestational weeks compared to the large intervals between gestational weeks can indicate that certain kinematic parameters (plantarflexion angle and angular velocity) were not statistically different, while the knee angle and angular velocity were statistically different between 24 w and 30 w. Therefore, doctors or scholars must be more specific in their future diagnosis and research.

In this study, the sample size of fetuses was small, and the standard deviation was large, but the trend was still obvious. No method exists to control the movement of the fetus; thus, collecting data is time-consuming. It is also expensive to recruit pregnant women to participate. The study involved pregnant women at 24, 27, and 30 weeks, but the kinematics parameters at 27 weeks were not statistically different from those at the other two gestational weeks. Therefore, later studies should increase the sample size and conduct longitudinal studies to find the kinematic characteristics of fetal gestational weeks.

## 5. Conclusions

In this study, we used simi motion to analyze the kinematic characteristics of knee extension and plantar flexion of fetuses at different gestational weeks. The results showed that 27 w and 30 w fetuses presented significantly smaller knee angles at the end of kicking motion when compared with 24 w fetuses, and the mean angular velocity of knee joint increased significantly at 30 w fetuses compared with 24 w fetuses. The novelty and findings from this study can be supplement as a part measurement of the motor development in fetuses and a good indicator for early diagnosis. In addition, this study also provides a foundation for subsequent further investigations.

## Figures and Tables

**Table 1:** Lower limb joint angle changes during kicking motion at 24, 27, and 30 gestational weeks (angle unit: degrees).

	Gestational Weeks			*p* Values, Mean Difference (95%CI)		
	24 Weeks	27 Weeks	30 Weeks	24 w/27 w	24 w/30 w	27 w/30 w
	Mean ± SD	Mean ± SD	Mean ± SD			
Knee						
Start angle	104.15 ± 1.20	102.28 ± 17.35	104.73 ± 29.69	0.97, 1.87 (−31.14, 34.89)	0.99, −0.58 (−59.72, 58.55)	0.98, −2.45 (−56.65, 51.75)
Final angle	174.76 ± 1.04	154.99 ± 4.49	154.89 ± 1.55	<0.01, 19.75 (9.47, 30.03)	<0.01, 19.86 (12.04, 27.68)	0.99, 0.11 (−9.81, 10.03)
Ankle						
Start angle	118.60 ± 2.44	86.36 ± 19.90	84.50 ± 41.32	0.09, 31.69 (−9.40, 72.80)	0.36, 33.56 (−52.52, 119.64)	0.99, 1.86 (−77.11, 80.84)
Final angle	150.34 ± 3.60	133.15 ± 26.13	133.46 ± 18.46	0.48, 17.19 (−36.60, 70.99)	0.30, 16.88 (−20.65, 54.41)	1.00, −0.31 (−51.01, 50.38)

Note: SD, standard deviation; CI: confidence interval.

**Table 2:** Lower limb joint angular velocity changes during kicking motion at 24, 27, and 30 gestational weeks (angular velocity: degrees/second).

	Gestational Weeks			*p*-Values, Mean Difference (95%CI)		
	24 Weeks	27 Weeks	30 Weeks	24 w/27 w	24 w/30 w	27 w/30 w
	Mean ± SD	Mean ± SD	Mean ± SD			
Knee						
Start angular velocity	19.95 ± 3.84	36.11 ± 20.01	42.70 ± 15.99	0.27, −16.15 (−50.41, 18.10)	0.08, −22.74 (−50.94, 5.45)	0.82, −6.58 (−40.46, 27.29)
Final angular velocity	22.83 ± 11.86	48.75 ± 30.14	23.37 ± 5.01	0.26, −25.92 (−75.86, 24.00)	0.99, −0.54 (−19.08, 17.99)	0.26, 25.38 (−26.33, 77.09)
Average angular velocity	35.04 ± 0.48	61.49 ± 23.44	50.22 ± 5.83	0.23, −26.45 (−78.16, 25.24)	0.02, −15.18 (−26.58, −3.78)	0.68, 11.27 (−38.75, 61.30)
Maximum angular velocity	75.13 ± 7.67	117.98 ± 28.44	121.42 ± 52.32	0.28, −42.84 (−135.85, 50.15)	0.29, −46.28 (−149.43, 56.86)	0.99, −3.43 (−107.08, 100.21)
Ankle						
Start angular velocity	9.86 ± 6.62	20.44 ± 10.55	15.98 ± 10.69	0.29, −10.58 (−30.78, 9.62)	0.62, −6.11 (−26.56, 14.32)	0.82, 4.46 (−18.57, 27.50)
Final angular velocity	18.02 ± 6.44	30.82 ± 20.20	31.22 ± 13.86	0.51, −12.79 (−52.57, 26.98)	0.29, −13.19 (−39.72, 13.34)	0.99, −0.39 (−39.43. 38.63)
Average angular velocity	33.23 ± 5.36	49.01 ± 13.00	55.94 ± 25.58	0.17, −15.77 (−40.85, 9.30)	0.31, −22.70 (−74.53, 29.12)	0.88, −6.93 (−55.72, 41.86)
Maximum angular velocity	104.77 ± 31.49	104.57 ± 21.61	131.04 ± 54.14	1.00, 0.19 (−60.64, 61.03)	0.69, −26.27 (−129.52, 76.98)	0.66, −26.46 (−131.17, 78.24)

Note: SD, standard deviation; CI: confidence interval.

## Data Availability

The data that support the findings of this study are available on reasonable request from the corresponding author. The data are not publicly available due to privacy or ethical restrictions.
